# Mammary Gland Pathology Subsequent to Acute Infection with Strong versus Weak Biofilm Forming *Staphylococcus aureus* Bovine Mastitis Isolates: A Pilot Study Using Non-Invasive Mouse Mastitis Model

**DOI:** 10.1371/journal.pone.0170668

**Published:** 2017-01-27

**Authors:** Jully Gogoi-Tiwari, Vincent Williams, Charlene Babra Waryah, Paul Costantino, Hani Al-Salami, Sangeetha Mathavan, Kelsi Wells, Harish Kumar Tiwari, Nagendra Hegde, Shrikrishna Isloor, Hesham Al-Sallami, Trilochan Mukkur

**Affiliations:** 1 School of Biomedical Sciences, Curtin Health Innovation Research Institute, Curtin University, Bentley, Perth, Western Australia, Australia; 2 College of Veterinary Sciences and Animal Husbandry, Central Agricultural University, Selesih, Aizawl, Mizoram, India; 3 Department of Medicine and Cell Biology, Albert Einstein College of Medicine, Bronx, New York, United States of America; 4 School of Pharmacy, Curtin Health Innovation Research Institute, Curtin University, Bentley, Perth, Western Australia, Australia; 5 School of Veterinary and Life Sciences, Murdoch University, Perth, Western Australia, Australia; 6 Ella Foundation, Genome Valley, Hyderabad, India; 7 Veterinary College, Karnataka Veterinary, Animal and Fisheries Sciences University, Bangalore, India; 8 School of Pharmacy, University of Otago, Dunedin, New Zealand; University Medical Center Utrecht, NETHERLANDS

## Abstract

**Background:**

Biofilm formation by *Staphylococcus aureus* is an important virulence attribute because of its potential to induce persistent antibiotic resistance, retard phagocytosis and either attenuate or promote inflammation, depending upon the disease syndrome, *in vivo*. This study was undertaken to evaluate the potential significance of strength of biofilm formation by clinical bovine mastitis-associated *S*. *aureus* in mammary tissue damage by using a mouse mastitis model.

**Methods:**

Two *S*. *aureus* strains of the same capsular phenotype with different biofilm forming strengths were used to non-invasively infect mammary glands of lactating mice. Biofilm forming potential of these strains were determined by tissue culture plate method, *ica* typing and virulence gene profile per detection by PCR. Delivery of the infectious dose of *S*. *aureus* was directly through the teat lactiferous duct without invasive scraping of the teat surface. Both bacteriological and histological methods were used for analysis of mammary gland pathology of mice post-infection.

**Results:**

Histopathological analysis of the infected mammary glands revealed that mice inoculated with the strong biofilm forming *S*. *aureus* strain produced marked acute mastitic lesions, showing profuse infiltration predominantly with neutrophils, with evidence of necrosis in the affected mammary glands. In contrast, the damage was significantly less severe in mammary glands of mice infected with the weak biofilm-forming *S*. *aureus* strain. Although both IL-1β and TNF-α inflammatory biomarkers were produced in infected mice, level of TNF-α produced was significantly higher (p<0.05) in mice inoculated with strong biofilm forming *S*. *aureus* than the weak biofilm forming strain.

**Conclusion:**

This finding suggests an important role of TNF-α in mammary gland pathology post-infection with strong biofilm-forming *S*. *aureus* in the acute mouse mastitis model, and offers an opportunity for the development of novel strategies for reduction of mammary tissue damage, with or without use of antimicrobials and/or anti-inflammatory compounds for the treatment of bovine mastitis.

## Introduction

Despite advances in diagnosis and management practices aimed at reducing the incidence of ruminant mastitis associated with the contagious pathogens, *Staphylococcus aureus* (*S*. *aureus*) is one of the major contagious causative agents of clinical and subclinical bovine mastitis worldwide causing significant economic loss to the dairy industry [1–3]. Reduced milk production due to mastitis is associated with irreversible mammary tissue damage in majority of the cases [4]. A wide array of virulence factors including surface-associated polysaccharide antigens including capsular polysaccharides (CP), teichoic acid (TA) and Poly-β-1, 6-N-acetylglucosamine (PNAG), MSCRAMM (microbial surface components recognizing adhesive matrix molecules), cytotoxins and enterotoxins are produced by *S*. *aureus*, which compromise host’s innate and adaptive immune systems [5, 6], resulting in the establishment of infection. *Staphylococcus aureus* enters the mammary gland, most likely via the orifice of chapped or injured teats [7]. Once the organism enters into the mammary gland, it adheres to epithelial cell receptors for bacterial adhesins [8] resulting in the production of virulence factors mentioned above and intracellular uptake of the small colony variants of *S*. *aureus*. Once the intra-mammary infection is established, damage to the mammary gland epithelial lining is initiated by ulceration and occlusion of lactiferous ducts, alveoli and infiltration of inflammatory cells in the parenchyma [4]. Mammary tissue damage is further compounded by various toxins and extracellular enzymes produced by *S*. *aureus*, including α, β, γ, and δ toxins, toxic shock syndrome toxin (TSST-1), enterotoxins, nuclease, coagulase, catalase, hyaluronidase, phosphatase, lipase, staphylokinase and proteases [9]. Some toxins act as super-antigens promoting the release of inflammatory cytokines, while others are leukocidins and cytolysins including haemolysins [10, 11]. Inflow of somatic cells, predominantly of polymorphonuclear (PMN) leukocytes into mammary gland is possibly due to breach of blood-milk barrier, worsens the damage to mammary epithelial cells owing to release of proteolytic enzymes and superoxide resulting in mammary tissue damage [12].

*Staphylococcus aureus* is capable of forming biofilms on both natural body surfaces and medical devices including indwelling catheters and prostheses resulting in acute or chronic infections [13]. Biofilm is a cluster of bacterial cells, extracellular matrix and water which is more tolerant to host defense mechanism and antibiotics [14] facilitating chronic infections [15–18]. The ability to produce biofilm by *S*. *aureus* in the mammary gland has been demonstrated both *in vitro* [19] and *in vivo* [20]. Development of biofilm by *S*. *aureus* has been proposed to occur in two steps. Regardless of the clinical manifestation of mastitis, acute/clinical or chronic/subacute/subclinical [21], the 1^st^ step is the adherence of *S*. *aureus* cells to mammary epithelial cells via microbial surface component recognizing adhesive matrix molecules (MSCRAMMs), including fibrinogen binding proteins (ClfA, ClfB), collagen binding protein (Cna), fibronectin binding proteins (FnBpA, FnBPB) and bone sialoprotein binding protein (BBP) [22–25]. Biofilm forming *S*. *aureus* can attach to mammary epithelium more efficiently than non-biofilm forming *S*. *aureus* [26, 27]. The 2^nd^ step involves multiplication, interaction and accumulation of adherent *S*. *aureus* cells embedded by bacterial extracellular matrix. The main constituent of extracellular matrix accredited for its role in the formation of biofilms by *S*. *aureus* is the polysaccharide intercellular adhesion (PIA) comprised of poly-β-1, 6-linked *N*-acetylglucosamine (PNAG) synthesized by *icaADBC* [28]. PNAG, a surface-associated polysaccharide is the most studied virulence factor associated with biofilm formation by *S*. *aureus*. However, *ica*-independent biofilm formation by *S*. *aureus* has also been reported, albeit in a small percentage of clinical isolates [29] suggesting potential importance of MSCRAMM and secretory proteins in replacing the function of PIA [30, 31]. Cytolytic toxins produced by *S*. *aureus* such as alpha (hla) and beta (hlb) have also been reported to be important in biofilm formation [32]. Alpha-toxin is required for cell-cell interactions during biofilm formation [10], whereas beta-toxin forms covalent cross-links to self to form insoluble nucleoprotein matrix in the presence of DNA in the staphylococcal biofilm [32]. Both alpha (α) and beta (β) toxins can directly damage milk-producing tissue by destruction of the mammary epithelial cells lining the teats and gland cisterns within the quarter. This eventually leads to establishment of deep-seated pockets of infection in the milk secreting cells (alveoli) and formation of scar tissue in acute mastitis, comparable to the formation of chronic wounds due to biofilm-mediated infection of the skin with this pathogen [33]. Other *S*. *aureus* proteins that potentially may be associated with biofilm formation include the immune evasion molecule, protein A [34], and biofilm-associated protein, Bap [35].

*Staphylococcus aureus* in biofilm is different from its planktonic state in various aspects including phenotypes [36], antibiotic resistance patterns [15] and innate immune responses [37]. More recently, it was demonstrated that *S*. *aureus* in biofilm induced stronger and differential immune responses against experimentally induced acute mastitis in mice than planktonic cultures [29]. Although many chronic infections are associated with bacteria in biofilm state as there is aggregation of colonies producing exopolysaccharide matrix offering resistance against phagocytosis and antibiotics [15], acute infection can also lead to the establishment of chronic carrier state. Baselga and co-workers (1993) compared slime producing (SP) versus non-slime producing (NSP) *S*. *aureus in-vitro* and *in-vivo*, and reported the NSP *S*. *aureus* strains to be more virulent than SP strains whereas the latter strains were more important in colonisation of mammary tissue [26]. Due to the ability of *S*. *aureus* in biofilm to induce inflammatory responses distinct from those induced by planktonic cultures [37, 38], it is important to know the extent of mammary tissue damage associated with infection of the mammary gland with strong biofilm forming versus weak or non-biofilm forming *S*. *aureus*. A pilot study by Breyne and co-workers [39] aimed at determining the differences between bovine associated coagulase-negative staphylococci (CNS) versus the laboratory *S*. *aureus* Newbould 305 strain. Bacterial inoculum containing low colony forming units (CFU) representing chronic or subclinical bovine mastitis in the non-invasive mouse model [40] resulted significantly lower bacterial growth of CNS versus the laboratory *S*. *aureus* strain. While *S*. *aureus* was reported to induce IL-6 and IL- 1β locally but not TNF-α, the CNS-infected mammary glands lacked strong cytokine host response [39]. This was in contrast to expression and induction/production of high levels of IL- 1β and TNF-α, with a low level of IL-6 in the plasma [41] in which the mouse mammary glands were infected with low CFUs of a clinical *S*. *aureus* isolate. However, no studies on the impact of biofilm formation on production of cytokines and mammary tissue damage were conducted. Our pilot study, presented in this communication, provides this information adding new knowledge in the area of bovine mastitis. Bovine mastitis in dairy cattle can manifest as clinical or acute, or subclinical/subacute, latter frequently leading to development of chronic mastitis [21]. Due to paucity of knowledge of the mammary gland pathology and cytokine profiles post-infection with strong biofilm-forming versus weak biofilm-forming *S*. *aureus*, this pilot study was carried out. Because of limited knowledge on the relative contribution of individual surface-associated antigens, such as polysaccharides (CP, TA, PNAG), MSCRAMM proteins and/or toxins to biofilm formation,*in vitro* or *in vivo*, it was decided to use *S*. *aureus* clinical isolates with the desired biofilm forming properties in this pilot study. The inflammatory cytokine response of lactating mice infected with large doses of strong versus weak biofilm-forming *S*. *aureus* to induce acute mastitis, suggested potential association of mammary tissue damage with high levels of TNF-α. Although IL-1β production was significantly greater in infected versus placebo mice also, there was no significant difference between the levels produced post-infection of the mammary glands with strong versus weak biofilm-forming *S*. *aureus*. The overall similarity of the cytokine profile induced by infection of the mammary gland with low [41] or high CFUs of *S*. *aureus* (this study) offers opportunities for development of treatment strategies aimed at eliminating or reducing mammary tissue damage in acute clinical bovine mastitis with or without the use of antibiotics and/or anti-inflammatory molecules.

## Materials and Methods

### Bovine *S*. *aureus* phenotypes

Two capsular polysaccharide-8 (CP8) positive *S*. *aureus* strains, isolated from mastitic milk of cows, with strong or weak biofilm forming potential, were used in this study (Table 1). These strains were selected from a collection of 154 strains, obtained through the courtesy of Professor Margaret Deighton, (RMIT University), Dr. Sharon de Wet (Queensland Biosecurity laboratory) and Dr. Justine Gibson (University of Queensland). These strains were collected from cows in dairy farms in Victoria and Queensland, Australia suffering from both clinical and subclinical mastitis. Selection of these strains was solely on the basis of *in vitro* biofilm forming ability [42] and presence or absence of biofilm-associated intercellular adhesin genes, *ica*A and *ica*D [6].

**Table 1:** Phenotypic and genotypic characteristics of *S*. *aureus* strains used in this study.

G Group	*S*. *aureus* strain	CP type	Biofilm value and grading (TCP method)	Presence of *ica* locus	Detectable MSCRAMM genes	Presence of toxin genes
1	*S aureus* 51	CP8	1.298 (Strong)	*icaA*, *icaD*	*cna*, *clfA*, *clfB*, *spa*,*isdA*,*isdB*,*sdrD*,*sdrE*	*hla*, *hlb*
2	*S aureus* 104	CP8	0.484 (Weak)	None	*cna*, *clfB*, *isdA*,*isdB*,*sdrD*,*sdrE*	*hla*, *hlb*

The gene encoding PVL was undetectable in both the strains used in this investigation.

### Capsular typing of *S*. *aureus* isolates

Capsular typing of the two *S*. *aureus* strains used in this investigation was carried out using molecular as well as serological methods as described elsewhere [43]. Briefly, genomic DNA was extracted from 2 strains of *S*. *aureus* using extraction kit (MO BIO laboratories, Inc, Carlsbad, CA). Conventional Polymerase Chain Reaction (PCR) was carried out using the cycling parameters for *cap*1, *cap*5 and *cap*8 described previously [43]. The primers used for typing *cap*1 are 5’-AGG TCT GCT AAT TAG TGC AA-3’ (forward) and 5’-GAA CCC AGT ACA GGT ATC ACC A-3’ (reverse) with an expected band size of 550bp. The primers used for typing *cap*5 and *cap*8 were 5′-GTC AAA GAT TAT GTG ATG CTA CTG AG-3′ (forward) and 5′-ACT TCG AAT ATA AAC TTG AAT CAA TGT TAT ACA G-3′ (reverse) for *cap*5 [42, 44] and 5′-GCC TTA TGT TAG GTG ATA AAC C-3′ (forward) and 5′-GGA AAA ACA CTA TCA TAG CAG G-3′ (reverse) for *cap*8 [43, 45], respectively. The expected band size for *cap*5 and *cap*8 were 881 bp and 1148 bp, respectively. Strain M, strain Newman and USA 400 and were used as positive control for *cap*1, *cap*5 and *cap*8 respectively, and LAC (USA 300) was used as negative control. For serological typing of *S*. *aureus* isolates, CP-specific antisera (CP 1, 2, 5 and 8) were produced using Quackenbush Swiss line 5 mice after gaining approval from the Animal Ethics Committee of Curtin University (Approval number: AEC_2011_65). The preparation of the vaccines for infection of mice and production of CP-specific sera were carried out according to the methods described elsewhere [46]. Strain M, strain Smith diffuse, strain Newman and USA 400 were used for production of CP1, 2, 5 and 8—specific sera, respectively. A slide agglutination test to determine the serotype of the four strains was carried out as described previously [46].

### Determination of biofilm forming potential of *S*. *aureus* isolates

#### Biofilm assay

Biofilm forming characteristics of the strains were determined using the Tissue Culture Plate method as described previously [42, 47].

#### Tissue culture plate method

*S*. *aureus* strains were inoculated in 200 μL of nutrient broth supplemented with 1% glucose in a 96-well sterile microtiter plate [48]. The plates were left overnight in a 37°C incubator to ensure adequate biofilm production. This was followed by assessment of their biofilm-forming property using the following method:

#### Crystal violet staining

This method, which measures the amount of biofilm attached to the polystyrene plate, was an adaptation of the procedures described elsewhere [47, 49]. *S*. *aureus* strains were grown to produce biofilm in a 96-well micro titre plate. The plates were washed with phosphate buffer saline (PBS) [pH 7.2] to remove planktonic or free-floating bacteria [49]. The biofilm was fixed at 55°C for 10 mins and stained with 2% crystal violet. Biofilms were de-stained with alcohol and read using a microplate reader at 570 nm. The assay was repeated three times and the data averaged. The arbitrary cut-off points for non-biofilm former, weak-biofilm former, intermediate and strong biofilm formers were 0.120 *A*_*570nm*,_ 0.130–4.00 x 0.120 *A*_*570nm*_, 4.10–5.90 x 0.120 *A*_*570nm*_ and greater than 6 x 0.120 *A*_*570nm*,_ respectively [50].

### ica typing of *S*. *aureus* isolates

The *ica* typing of the isolates was performed as per the method described elsewhere [51]. Briefly, The primers used for typing *ica*A [52] were 5’-CCTAACTAACGAAAGGTAG-3’ (forward) and 5’-AAGATATAGCGATAAGTG C-3’ (reverse) and 5’-AAACGTAAGAGAGGTGG-3’ (forward) and 5’-GGCAATATGATCAAGATAC-3’ (reverse) for *ica*D [52] typing. The expected band size for *ica*A and *ica*D were 1315bp and 381bp, respectively. The PCR cycles were run at 95°C for 5mins, 95°C for 30secs, Tm 48°C (*ica*A) and 47°C (*ica*D) for 30secs, 72°C for 45secs with 30 cycles and final extension at 72°C for 10 mins. Gel electrophoresis was carried out using 1.5% agarose gel in 1x TAE buffer and visualizing under UV trans-illuminator. The positive control used for this study was USA 400 strain which was positive for both *ica*A and *ica*D.

### Detection of different virulence genes of *S*. *aureus* using PCR

Conventional PCR was carried out to detect MSCRAMM associated and toxin genes of the two *S*. *aureus* strains isolated from both clinical and subclinical bovine mastitis cases in Australia. The primers, Tm, for all the MSCRAMM-encoding including *icaA icaD* (intercellular adhesion molecule, PNAG), *cna (*collagen-binding adhesion protein), *clfA* (clumping factor A), *clfB* (clumping factor B), *spa* (protein A), *fnbpA* (fibronectin-binding protein A), *fnbpB* (fibronectin-binding protein B), *bbp* (bone sialoprotein binding protein), *isdA* (iron regulated protein A), *isdB* (iron regulated protein B), *sdrD* (serine-aspartate repeat proteins D), *sdrE* (serine-aspartate repeat proteins E) and *bap* (biofilm-associated surface protein) and cytolytic toxin genes (*hla* (haemolysin A), *hlb* (haemolysin B), *pvl* (panton-valentine leukocidin) of the two *S*. *aureus* strains used in this study have been described elsewhere [6, 51].

### Infection of mammary gland

#### Preparation of bacterial inocula

The *S*. *aureus* strains were grown on Mueller Hinton (MH) agar plates at 37°C for 18h. The organisms were washed from the plates using 20 ml of isotonic saline and suspended in isotonic saline to give a final viable bacterial count of 4 x 10^11^ ml^-1^ [53].

#### Animals

All animal work described in this investigation was approved by the Animal Ethics Committee of Curtin University (Approval number: AEC_2012_14) prior to commencement of the experiment. The mice were used for the study ensuring compliance with the Western Australian Animal Welfare Act 2002. A total of 12 Balb/c first-pregnancy mice, in three groups of 4 mice each were used for the experiment. Pups were removed from the lactating mice when they were between 5–15 days old, approximately 1h prior to infection of the mammary glands of mice. The pups were euthanized and not allowed to suckle after inoculation of the mammary gland with the *S*. *aureus* strains or normal saline in case of the control group of mice.

#### Method of infection of the mammary gland

Inoculation of the different *S*. *aureus* phenotypes into mammary glands was carried out using a modification to the procedure described previously [54]. The modification involved inoculation of *S*. *aureus* strains directly into the mammary duct without making any incision or scraping of the superficial layer of the mammary gland with a scissors or scalpel. Briefly, mice were anaesthetised using 100 mg kg^-1^ ketamine and 10 mg kg^-1^ xylazine administered by the intraperitoneal route and laid in the supine position. The area surrounding the 5^th^ pair of mammary glands (L5 and R5) was disinfected with 70% ethanol. A binocular dissecting microscope was used to locate the duct orifice of the teat. While controlling the teats with sterile forceps, 0.05 ml of bacterial suspension equivalent to 2x 10^10^ CFU *S*. *aureus* was injected using a blunt smooth 31-gauge hypodermic needle to a depth of not more than 4mm. The infection was allowed to progress for 48 h and the mice were observed at six-hour intervals to assess development of macroscopic clinical signs of infection. All animals were administered one dose of Buprenorphine hydrochloride (0.05–0.1 mg kg^-1^) subcutaneously pre-operation so that anticipated pain relief was available for up to 12 h post bacterial inoculation. A control group of mice was injected with normal saline using the same procedure.

### Post-inoculation examination

#### Macroscopic examination

The mammary glands of mice were examined for the development of mastitis. The clinical signs of mastitis observed were redness, swelling and discoloration of mammary gland, and extrusion of exudate with or without squeezing of the mammary gland. Monitoring of mice for morbidity and mortality was carried out at 6 hourly intervals up to 48h. The level of clinical signs was graded as 0 (no macroscopic changes), + (low) grade, ++ (medium grade) and +++ (severe grade) based on the observed clinical features.

Mice were euthanized 48h after infection, and investigated for the extent of infection by determining the bacterial load [53] as described below, and the mammary gland tissue collected for estimating the level of inflammation and histological changes [53].

#### Bacteriological procedures for mammary glands and blood

At post-mortem examination, the L5 mammary glands from control and test mice were collected and individually ground in sterile Griffith’s tubes containing 2 ml of sterile normal saline. The homogenates from the mammary glands were subjected to serial tenfold dilutions and inoculated on Baird Parker (BP) agar plates by the spread plate method and incubated at 37°C for 48h, followed by determination of colony counts of *S*. *aureus* per mammary gland.

Blood samples obtained by cardiac puncture were inoculated on BP agar plates by streaking to obtain isolated colonies and incubated at 37°C for 48 h.

### Histological procedures

#### Mammary gland and blood

After aseptically shaving the hair around the gland the R5 mammary glands were collected for histological examination. The R5 glands were fixed in 10% neutral buffered formalin for 24 h, processed on an automatic tissue processor and embedded in paraffin wax. Sections were cut at 4 μm thickness at three levels and stained by the Haematoxylin and Eosin stain [55]. An additional section was stained for bacteria using Gram Twort Method [56].

Blood smears were prepared following standard procedure and stained by the Diff-Quik method [57].

#### Grading of histological changes observed in mammary glands

**Fig 1:**
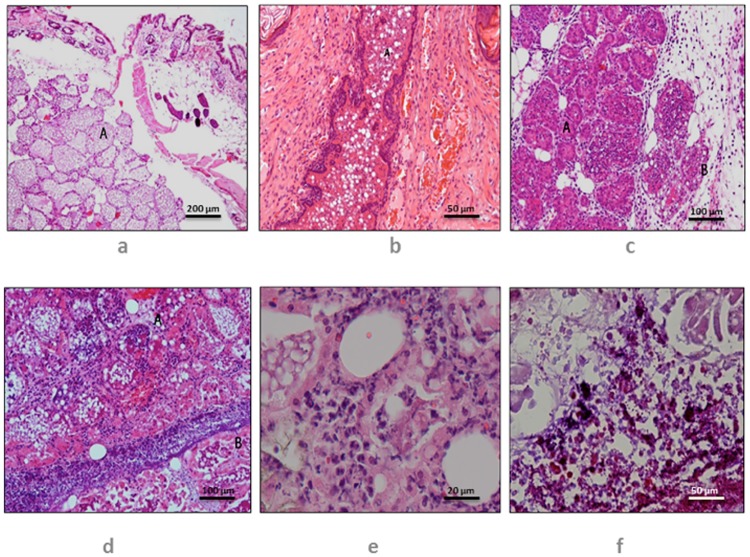
Histopathological changes observed in mouse mammary tissue. Control mouse showing absence of inflammatory response (Level 0) in mammary tissue (A) to inoculation of sterile normal saline, H&E x100, bar, 200 μm (Fig 1a); Level 1 inflammatory response induced in the mouse teat by inoculation of *S*. *aureus* via the mammary duct (A), characterized by acute neutrophil rich response in the intraductal exudate, H&E x400, bar, 50 μm (Fig 1b); Level 2 inflammation of the mammary glands (A) Infiltrate of acute inflammatory cells, predominantly neutrophils, in supporting connective tissue, with intraluminal organisms, H&E x200, bar, 100 μm (Fig 1b); Level 2 inflammation of the mammary tissue post-inoculation of mammary gland with *S aureus*, characterized by infiltration of acute inflammatory cells, predominantly neutrophils (A), in supporting connective tissue and intraluminal space, H&E x200, bar, 100 μm (Fig 1c); Level 3 inflammation of mammary tissue post-inoculation of mammary gland with *S*. *aureus*, characterized by marked acute neutrophil rich infiltrate (A) with tissue necrosis (B), H&E x200, bar, 100 μm (Fig 1c); Level 3 inflammation of the mammary tissue post-inoculation of mammary gland with *S*. *aureus*, characterized by marked acute neutrophil-rich infiltrate (A) with tissue necrosis (B), H&E x200, bar, 100 μm (Fig 1d); Level 3 inflammation of the mammary tissue post-inoculation of mammary gland with *S*. *aureus*, characterized by neutrophil-rich inflammatory exudate, H&E x1000, bar, 20 μm (Fig 1e); Level 3 inflammation of the mammary tissue post-inoculation of mammary gland with *S*. *aureus*, characterized by presence of Gram-positive bacteria and neutrophil-rich inflammatory exudate, Gram Twort x400, bar, 50 μm (Fig 1f).

The histopathological changes observed in mammary glands of mice, infected with different strains of *S*. *aureus*, were graded as Level 0 [no inflammatory reaction post-inoculation with PBS (Fig 1a)], Level 1 [minimal inflammatory response in mammary tissue post-inoculation with *S*. *aureus* (Fig 1b)], Level 2 [Moderate inflammation in peri-mammary and intramammary tissue post-inoculation with *S*. *aureus*, with intra luminal organisms observed (Fig 1b and 1c), and Level 3 [Marked inflammatory cell infiltration into mammary tissue post-inoculation with *S*. *aureus* with evidence of tissue degeneration including necrosis (Fig 1d to 1f)].

### Quantification of inflammatory cytokines

Quantification of inflammatory cytokines, IL-1β and TNF-α in serum samples of mice was performed by using BD Cytometric Bead Array (CBA) Mouse/Rat soluble protein Master Buffer Kit (BD Biosciences), USA. Preparation of Mouse/Rat soluble protein flex set standards, capture beads and detection reagents were performed as per the standard protocol provided with the reagent kit. Briefly, 50μL of Mouse/Rat soluble protein flex set standard dilutions ranging from 1:2 to 1:256 and one negative control containing only assay diluent was prepared. To 10μL of each unknown serum sample, 10μL of each capture bead and mixed PE detection reagent was added. Tubes were incubated at 4°C for one hour each after addition of capture beads and PE detection reagent protected from light. Following incubation, 200μL of wash buffer was added to each tube and centrifuged at 200g for 5min. The supernatant was aspirated carefully and discarded. The remaining pellet was reconstituted using 200μL of wash buffer and analysed using Attune Acoustic Focussing Flow Cytometer using the FlowJo software.

### Statistical analysis

To compare the IL-1β and TNF-α levels, bacterial loads between groups of mice infected with strong biofilm and weak biofilm forming *S*. *aureus* phenotypes, student’s t-test was performed using Microsoft Excel. Statistical significance was set at p < 0.05.

## Results

### Determination of biofilm forming potential of *S*. *aureus* isolates

#### Tissue culture plate (TCP) method

Between the two *S*. *aureus* strains selected for this study, *S*. *aureus* strain 51 was strong biofilm former *in vitro* whereas *S*. *aureus* strain 104 was a weak biofilm former *in vitro* (Table 1).

#### ica typing of *S*. *aureus* isolates

*Staphylococcus aureus* strains 51 harboured the *ica*A and *ica*D genes encoding production of PNAG whereas these genes were not detectable in *S*. *aureus* strain 104.

#### Detection of different MSCRAMM-encoding genes of *S*. *aureus* using conventional PCR

The virulence-associated genes detected in the two bovine *S*. *aureus* strains are shown in Table 1. Both *S*. *aureus* strains harboured genes encoding alpha and beta toxin. *S*. *aureus* 51 carried *icaA icaD*, *cna*, *clfA*, *clfB*, *spa*, *isdA*, *isdB*, *sdrD* and *sdrE* MSCRAMM genes and also genes encoding alpha and beta haemolysins. *S*. *aureus* 104 was found to carry *cna*, *clfB*, *isdA*, *isdB*, *sdrD* and *sdrE* genes, the gene encoding *spa* and *clfA* being undetectable (Table 1).

#### Macroscopic examination of mammary glands

The control group showed no gross/macroscopic pathological change in the inoculated glands. On the other hand, all the test mice showed varying degrees of change in the gross appearance of mammary glands (Table 2).

**Table 2:** Clinical signs observed in mice infected with strong versus weak-biofilm forming *S*. *aureus* strains by intra-mammary route (observations up to 48 hours post inoculum).

Time post inoculation	*S*. *aureus* 51	*S*. *aureus* 104	Control
6h	0	0	0
12h	+	+	0
18h	++	+	0
24h	++	+	0
30h	+++	++	0
36h	+++	++	0
42h	+++	++	0
48h	+++	++	0

Clinical features used to make a judgement on the macroscopic pathology of the mammary glands inoculated with S. aureus strains include redness, swelling, and discolouration of mammary gland, exudate, morbidity and mortality. Macroscopic pathological changes were assigned scores as no change (0) low grade (+), medium grade (++) and severe grade (+++). The criteria used for grading of clinical signs are shown Supporting Information (S1 Table).

#### Bacterial load and histopathological changes of mammary gland

The bacterial loads in the mammary glands as a measure of proliferation of *S*. *aureus* at the infection site and the associated histopathological grade observed in the mammary tissue are shown in Table 3 and Fig 1. The bacteriological load study demonstrated that mice (M) infected with encapsulated *S*. *aureus* strains with *in vitro* strong biofilm forming ability (*S*. *aureus* 51) had higher bacterial count than the encapsulated *in vitro* weak biofilm producing strain (*S*. *aureus* 104). Statistical analysis of the bacterial burden of the mammary glands of mice infected with strong biofilm-forming versus weak biofilm-forming *S*. *aureus* strains revealed significant differences in the bacterial load between the different groups (P < 0.001).

**Table 3:** Total viable counts of *S*. *aureus* recovered from mammary glands 48 h post-harvest and the grade of histopathological changes.

Group	*S*. *aureus* phenotype	Total number of mammary glands investigated	Log average no. of bacteria (CFU) recovered from mammary glands	Grades for histopathological changes			
				M1	M2	M3	M4
1	*S*. *aureus* 51	4	8.0806 ± 0.0050	3	3	3	3
2	*S*. *aureus* 104	4	7.5260 ± 0.0189	2	2	2	2
3	Control (Normal saline)	4	-	0	0	0	0

The detailed data pertaining to this experiment is shown as Supporting Information (see S2 Table)

Histopathological studies showed that mice infected with encapsulated *S*. *aureus* with strong biofilm forming characteristics (*S*. *aureus* 51) produced severe mastitis lesions characterised as level 3 lesions, showing a profuse inflammatory infiltrate with severe tissue damage including necrosis in mammary tissue. On the other hand, *S*. *aureus* strain 104 with weak biofilm forming ability demonstrated only level 2 histopathological changes (Table 3).

#### Bacteriology of blood and and histopathology of liver, lung and spleen

The culture of blood and organs (liver, lung and spleen) in BP agar plates was negative for *S*. *aureus* consistent with no evidence of systemic infection. No overt microscopic evidence of inflammation was observed in tissue sections of lung, liver and spleen from any of the mice as judged by normal histological appearance (data not shown).

#### Histopathology of mammary glands pre- or post-infection with biofilm forming *S*. *aureus*

No evidence of inflammatory response was recorded in mammary tissue of control mice which were inoculated with sterile normal saline. The mammary tissue of mice infected with *S*.*aureus* 51 demonstrated level 3 inflammation with marked neutrophil rich infiltration and tissue necrosis. The mammary tissue of all the mice infected with *S*. *aureus* 104 showed level 2 inflammation characterised by acute inflammatory cells, predominantly neutrophils, in supporting connective tissue and intraluminal spaces (Fig 1).

#### Quantification of inflammatory cytokines

Quantification data of inflammatory cytokines, IL-1β and TNF-α is presented in Table 4. The result showed that there was no significant difference in the level of IL-1β produced by strong biofilm forming versus the weak biofilm forming *S*. *aureus* strain. However, the levels of TNF-α were significantly higher (p<0.05) in the sera of mice inoculated with strong biofilm forming *S*. *aureus* strain 51 versus infection with the weak biofilm forming *S*. *aureus* strain 104.

**Table 4:** Detection of IL-1β and TNF-α levels in serum samples of mice 48 h post-infection.

Group	*S*. *aureus* phenotype	IL-1β±SE (Pg/mL)	TNF-α±SE (Pg/mL)	Histopathology grade
1	*S*. *aureus* 51	3.641±0.35	13.00±0.50	3
2	*S*. *aureus* 104	3.19±0.59	8.85±1.97	2
3	Control	0.00	0.00	0

TNF-α levels between groups of mice inoculated with strong biofilm (*S*. *aureus* 51) versus weak-biofilm forming *S*. *aureus* (*S*. *aureus* 104) strains showed significantly higher (p < 0.05) differences. Detailed data pertaining to this experiment is presented as Supporting Information (S3 Table)

## Discussion

Biofilm formation by *S*. *aureus* has been studied extensively with respect to methods of detection, potential role in chronicity of infection and resistance to antibiotics and innate immune defences for both human and animal infectious diseases [13–15, 38, 41, 58]. Demonstrated ability of *S*. *aureus* biofilm to resist innate immune defences against intra-mammary infections (IMI) in the last few years [31, 37, 59, 60] and the difficulty encountered in treating such infections with antibiotics [9] has highlighted the urgency of developing effective vaccines against bovine mastitis. This also highlights the urgency of discovering ways to disperse the biofilms for improvement in the efficacy of the available antibiotics in the treatment of intra-mammary infections. Potential reasons underpinning mammary tissue damage due to *S*. *aureus* infection have been reported previously [4, 61]. However, limited studies have been reported on the potential impact of biofilm formation by *S*. *aureus* on the integrity of the mammary tissue post-infection with the pathogen [26, 62]. Most studies have been confined to characterization of the inflammatory immune responses to porcine dermal explants [63], murine tibial implants [64], nasal explant model [65] and prosthetic implant infections [64, 66]. Baselga and co-workers (1993) reported slime producing (SP) *S*. *aureus* isolates from ruminant mastitis cases to exhibit higher colonisation capacity than the non-slime producing (NSP) variant of the same strain obtained by repeated subculturing on Congo red agar at a medium frequency [26]. The NSP variants were shown to have higher virulence than the SP *S*. *aureus* revertants as measured by moderate to severe clinical signs and inflammatory lesions in mammary glands of lactating ewes at 48 h post-infection. It was hypothesized that the SP isolates of *S*. *aureus* were responsible for attachment to mammary epithelia and colony formation, whereas the NSP *S*. *aureus* variant led to the development of severe mastitis. Since some NSP strains can produce slime *in vivo* as demonstrated by immunoperoxidase staining of mammary tissue [26], it is important to obtain information on the strength of the biofilm formation of SP versus NSP strains cultured *in vitro* as well as for isolates cultured from infected mammary tissues for confirmation of the stated hypotheses.

In our study, the main aim was to compare the pathology subsequent to infection of the mammary glands with a high dose of mammary infection (2x 10^10^ CFU/0.05 ml) [53] of strong biofilm versus weak-biofilm producing clinical *S*. *aureus* strains. Development of acute mastitis was measured by the level of mammary tissue damage based upon clinical manifestations, histopathology, and the production of inflammatory cytokines. The only differences between the 2 isolates used in this investigation are the detection of *ica*A and *ica*D genes encoding production of PNAG, previously reported to be associated with biofilm formation [67, 68] and the *spa* and *clfA* genes encoding the production of protein A and *clfA* clumping factor A respectively in the strong biofilm forming strain 51, which were undetectable in the weak biofilm forming *S*. *aureus* isolate/strain 104 (Table 1). The strong biofilm forming property of *S*. *aureus* strain 51 may also be attributable, in part, to the presence *spa* and *clfA* genes encoding protein A and clumping factor A respectively. The relative contribution of protein A and ClfA to colonisation of mammary glands warrants further investigations. Other genes that have all been reported previously to contribute to biofilm formation and were detectable in both the isolates included *cna*, *clfB*, *isdA*, *isdB*, *sdrD*, *sdrE*, *hla and hlb* [22, 23, 32, 69–71]. Had information about the relative contribution of each of the genes detected in this investigation been available, it may have been possible to confirm the biofilm forming property of the strains used in this investigation by constructing isogenic mutants. However, given the recent report of major potential contribution of PNAG to biofilm formation [72], the weak biofilm forming *S*. *aureus* strain 104 with no detectable *icaA* and *icaD* genes, appears to be a reasonable choice for this investigation. It is also to be noted that all the *S*. *aureus* strains used in this study were negative for the gene encoding bap, which has been reported to facilitate formation of biofilm in chronic bovine mastitis [15].

The bacteriological load study demonstrated significantly higher bacterial counts in the mammary glands of mice infected with strong biofilm forming *S*. *aureus* strain 51 than those infected with the weak biofilm forming strain 104. Although severity of mastitis has been reported to vary from cow to cow, level of bacterial load may also play a role in the extent of mammary tissue damage. Significantly higher bacterial load in the mammary glands of mice infected with strong biofilm forming *S*. *aureus* versus those infected with weak biofilm former may be associated with the ability of the former strains to attach and colonize mammary epithelia more efficiently, a perquisite for manifestation of clinical mastitis. This suggestion is supported by previous studies [73] in which significant correlation between the bacterial load and severity of mastitis within 6 h of intramammary inoculation of *Escherichia coli* into mammary gland of cows was reported.

Histopathological analysis is an important and widely used tool to assess mammary tissue damage incurred by bacterial invasion of mammary gland [4, 74]. Extensive PMN infiltration, reduced alveolar luminal space, necrosis of mammary tissue and replacement of damaged secreting mammary tissue with non-secretory mammary tissue are the characteristic histopathological findings in bovine mastitis caused by *S*. *aureus* [75–77]. PMN plays a vital role in host defence mechanism against *S*. *aureus* invasion of mammary gland [78]. However, excessive levels of PMN, which may rise as high as 90% in bovine mastitis cases [79], can potentially damage mammary epithelia by respiratory burst and degranulation leading to release of granular enzymes [60]. In the present study, inflammation in peri-mammary and intra-mammary tissue with intra-luminal organisms and marked inflammatory cell infiltration into mammary tissue with evidence of tissue degeneration including necrosis were observed. Given the production of greater mammary tissue damage (grade 3 histopathological changes) by the strong biofilm-producing *S*. *aureus* strains versus grade 2 lesions by the PNAG-independent weak biofilm-producing strain in this study, there appeared to be an association between the strength of the biofilms produced by *S*. *aureus* and damage to the mouse mammary tissue (Table 3, Fig 1d).

The vital mediators of inflammatory process during IMI are the cytokines [80]. Many investigators have reported higher concentrations of IL-1 [81] and TNF- α [82] in milk and plasma during mastitis potentially mediating local and systemic inflammatory responses. The level of TNF-α in serum has been reported to be highly elevated in cases of acute clinical mastitis [83] resulting in activation and migration of neutrophils [84]. Nevertheless, no studies on the role of these mediators in mammary tissue damage caused by strong versus weak biofilm-forming *S*. *aureus* have been performed. TNF-α was reported to induce apoptosis in bovine endothelial cells [85] and human mammary gland epithelial cells [86]. In our study, the level of TNF-α was significantly higher (p<0.05) in mice inoculated with the strong biofilm forming *S*. *aureus* than the weak biofilm forming strain even at 48 h post-infection indicating an important role for TNF-α in the observed mammary tissue damage in the mouse mastitis model (Table 4). An earlier investigation [87] reported a potential protective role of TNF-α against *S*. *aureus*-associated mastitis provided it was administered by the i/mam route prior to infection (4 to 0 hours) of the mouse mammary gland, accompanied by administration of ciprofloxacin or pirlimycin 1 hour post-infection of the mammary gland. However, no information on the potential association of *S*. *aureus* biofilms with inflammatory cytokines and mammary tissue damage post-infection was reported. In contrast, Breyne and co-workers (2015) reported that infection of the mouse mammary gland with *S*. *aureus* resulted in local induction of IL-1β and IL-6 but not TNF-α [39]. However, these authors found that mice inoculated with CNS lacked a strong cytokine response although IL-1β was produced locally. Clearly, an analysis of the cytokine profile post-infection with the biofilm forming Newbould strain 305, which is genotypically different from a virulent field isolate, RF122, a strain representative of the major clone involved in severe bovine mastitis worldwide [88], using a dose high enough to produce acute mouse mastitis warrants further investigations. Similarly, further studies on the extent of mammary tissue damage post-infection of the mammary glands with doses low enough to induce subclinical or chronic mouse mastitis by the strong versus weak biofilm forming strains used in this investigation and association/correlation with the levels inflammatory cytokines produced, warrants further investigations.

In summary, the extent of tissue damage in the mammary gland of mice infected with strong biofilm forming *S*. *aureus* was found to be significantly greater than that associated with infection with weak biofilm forming *S*. *aureus* in this pilot study. It was concluded that the severe tissue damage in mammary glands of mice infected with *S*. *aureus* might be due to the strength of biofilm formation by the pathogen and production of the inflammatory cytokine, TNF-α, mediating the influx of phagocytic cells in the mammary gland [89]. However, the limitation of this study is the lack of availability of bovine mastitis-associated clinical *S*. *aureus* isolates that are genotypically and phenotypically identical, with the only difference comprising the strength of biofilm formation. Although beyond the scope of this study, it would be interesting to determine the extent of mammary tissue damage caused by a triple deletion mutant of the strong biofilm forming strain 51, in which the genes encoding protein A (*spa*), clfA (*clfA*) and PNAG (*icaA*, *icaD*) have been deleted, with a view to confirming their potential contribution to the strength of biofilm formation and associated mammary gland pathology. Mouse mastitis model, although requiring larger number of organisms for establishment of acute mammary gland infection, has been considered to be a viable alternative to dairy cattle for cost considerations and similarities between mice and cows because of the locale of mammary glands in the inguinal region [90] despite differences in the predominance of different immunoglobulin isotypes in the serum or milk of mice, depending upon the nature of the pathogen viz., extracellular versus intracellular.

The next phase of this study is to determine the attachment potential, a pre-requisite for biofilm formation [72], of the *S*. *aureus* strains used in this study for the mammary gland epithelial cells *in vitro* using bovine mammary epithelial cells (MAC-T), followed by *in vivo* studies using the non-invasive mouse mastitis model described in this investigation. Furthermore, because of the unlikelihood of compromising the cutaneous immune system [91], the non-invasive mouse mastitis model should be considered as a preferred alternative to the invasive mouse mastitis model, in which the skin covering the surface of the mammary gland is scraped off using a scalpel or scissors [27, 54], for assessment of the biofilm dispersing potential of biofilm disrupting molecules, including quorum quenching compounds, for discovery of novel anti-biofilm antimicrobials and anti-inflammatory molecules/compounds, notwithstanding its’ cost-effectiveness in the evaluation of novel vaccine candidates against bovine mastitis.

## Supporting Information

S1 Table Criteria used for assignment of grades based on the extent of inflammation in infected versus control mice at 48 hours post-infection. Assignment of zero (0) denotes no inflammation, + denoted low grade, ++ denoted medium grade and +++ severe inflammation using redness, swelling, discoloration of mammary gland, presence of exudate in the teat, morbidity and mortality.
(DOCX)

S2 Table Raw data of total viable counts (CFU/infected mammary gland) of strong versus weak biofilm forming *S*. *aureus* recovered from mammary glands at 48 hours post-infection. A total of 4 mice were infected with each *S*. *aureus* strain and euthanized at 48 hours post-infection for collection of mammary glands and total bacterial load/mammary gland obtained in each mouse.
(DOCX)

S3 Table Raw data of IL-1β and TNF-α levels (Pg/mL) in serum samples of mice infected with *S*. *aureus* at 48 hours post-infection. Blood samples were collected post-euthanasia at 48 hours post-infection **for** collection of sera and cytokines estimated for each experimental mouse.
(DOCX)
