# Supplementary material for: Mammary Gland Pathology Subsequent to Acute Infection with Strong versus Weak Biofilm Forming Staphylococcus aureus Bovine Mastitis Isolates: A Pilot Study Using Non-Invasive Mouse Mastitis Model
Source: PLoS One. 2017 Jan 27;12(1):e0170668. doi: 10.1371/journal.pone.0170668 (PMC5271311; doi:10.1371/journal.pone.0170668)
Supplement: S1 Table — Assignment of zero (0) denotes no inflammation, + denoted low grade, ++ denoted medium grade and +++ severe inflammation using redness, swelling, discoloration of mammary gland, presence of exudate in the teat, morbidity and mortality. (DOCX) [file pone.0170668.s001.docx]

**S1 Table. Criteria used for assignment of grades based on the extent of inflammation in infected and control mice**

| **Grade*** | **Criteria used for assignment of severity of inflammation** | | | | | |
| --- | --- | --- | --- | --- | --- | --- |
|  | **Redness** | **Swelling** | **Discoloration of mammary gland** | **Exudate** | **Morbidity** | **Mortality** |
| 0 | − | − | − | − | − | − |
| + | + | − | − | − | − | − |
| ++ | + | + | - | − | + | − |
| +++ | + | + | + | + | + | ± |
