# Supplementary material for: Mammary Gland Pathology Subsequent to Acute Infection with Strong versus Weak Biofilm Forming Staphylococcus aureus Bovine Mastitis Isolates: A Pilot Study Using Non-Invasive Mouse Mastitis Model
Source: PLoS One. 2017 Jan 27;12(1):e0170668. doi: 10.1371/journal.pone.0170668 (PMC5271311; doi:10.1371/journal.pone.0170668)
Supplement: S2 Table — A total of 4 mice were infected with each S. aureus strain and euthanized at 48 hours post-infection for collection of mammary glands and total bacterial load/mammary gland obtained in each mouse. (DOCX) [file pone.0170668.s002.docx]

**S2 Table. Raw data of total viable counts (Colony Forming Units [CFU] /infected mammary gland) of strong versus weak biofilm forming *S. aureus* recovered from mammary glands at 48 hours post-infection**

| ***S. aureus* strain and mouse #** | **Raw value** | **x80x10^4^**  **CFU** | **log10**  **CFU** | **Average CFU ± SD** | **Average CFU ± SEM** |
| --- | --- | --- | --- | --- | --- |
| 51.1 | 150 | 1.2E+08 | 8.079181 | 8.0806  **±** 0.0050 | 8.0806  **±** 0.0025 |
| 51.2 | 153 | 1.22E+08 | 8.087781 |  |  |
| 51.3 | 150 | 1.2E+08 | 8.079181 |  |  |
| 51.4 | 149 | 1.19E+08 | 8.076276 |  |  |
| 104.1 | 43 | 34400000 | 7.536558 | 7.5260  **±** 0.0189 | 7.5260  **±** 0.0095 |
| 104.2 | 44 | 35200000 | 7.546543 |  |  |
| 104.3 | 40 | 32000000 | 7.505150 |  |  |
| 104.4 | 41 | 32800000 | 7.515874 |  |  |
