# Supplementary material for: Mammary Gland Pathology Subsequent to Acute Infection with Strong versus Weak Biofilm Forming Staphylococcus aureus Bovine Mastitis Isolates: A Pilot Study Using Non-Invasive Mouse Mastitis Model
Source: PLoS One. 2017 Jan 27;12(1):e0170668. doi: 10.1371/journal.pone.0170668 (PMC5271311; doi:10.1371/journal.pone.0170668)
Supplement: S3 Table — Blood samples were collected post-euthanasia at 48 hours post-infection for collection of sera and cytokines estimated for each experimental mouse. (DOCX) [file pone.0170668.s003.docx]

**S3 Table: Raw data of IL-1β and TNF-α levels (Pg/mL) in serum samples of mice infected with *S. aureus* at 48 hours post-infection**

| ***S. aureus* strain and mouse #** | **IL-1β**  **(pg/mL)** | **Average level (pg/mL)± SD** | **Average level (pg/mL)± SEM** | **TNF-α**  **(pg/mL)** | **Average level (pg/mL) ± SD** | **Average level (pg/mL)± SEM** |
| --- | --- | --- | --- | --- | --- | --- |
| 51.1 | 2.720 | 3.6405 **±** 0.6937 | 3.6405 **±** 0.3469 | 12.3453 | 12.9858 **±** 1.0049 | 12.9858 **±** 0.5024 |
| 51.2 | 3.964 |  |  | 12.0443 |  |  |
| 51.3 | 3.543 |  |  | 14.2673 |  |  |
| 51.4 | 4.335 |  |  | 13.2863 |  |  |
| 104.1 | 4.516 | 3.1918 **±** 1.1739 | 3.1918 **±** 0.5869 | 5.5773 | 8.85 **±** 3.9323 | 8.85 **±** 1.9661 |
| 104.2 | 1.808 |  |  | 10.1423 |  |  |
| 104.3 | 2.740 |  |  | 13.8433 |  |  |
| 104.4 | 3.703 |  |  | 5.8373 |  |  |
